# Our New York Letter

**Published:** 1889-07

**Authors:** M. B. Hutchins

**Affiliations:** New York


### OUR NEW YORK LETTER.

NEW YORK, June, 1889.

### DERMATOLOGICAL.

Recently, at the New York Skin and Cancer Hospital, Dr. L. D. Bulkley ordered a number of hard "tubercles" in a case of chronic eczema "touched" with pure carbolic acid. It was thoroughly done, and the lesions rapidly divided. Duhring only mentions the remedy as useful in lotion or ointment, and more for the purpose of relieving the itching than as a curative remedy. Crocker, on Diseases of Skin; Robinson and Bulkley in their "Manuals," and Hans von Hebra, in his work, "Die Kraukhaften Veränderungen der Haut," etc., have no reference to this use of carbolic acid.

In a previous letter I mentioned an eruption supposed to have been produced by salicylate of sodium. I have since learned that the eruption appeared while the man was taking a "rhubarb and soda mixture," and that there was a recurrence upon re-administration of the prescription; the eruption consequently was attributed to "rhubarb and soda." No article available on the subject of drug eruptions mentions either of the above as producing an eruption of any kind.

A man having a peculiar discoid or circumscribed outbreak of lesions averaging the size of a silver half-dollar, marked by pigmentation or close, adherent, dark scales, was given ext. ergot. fluid. ʒss t. i. d., increasing dose gtt. v. per day until gtt. lxxv were reached. No local treatment. Lesions slowly disappeared or became pigmented spots, as the older ones, but there is doubt as to the effect of the ergot. Recently the old discs of disease became the seat of a number of small vesico-pustules which, however, disappeared in a few days under carbolized vaseline. Disease was diagnozed as a circumscribed eczema by Dr. Bulkley, while his assistant, Dr. G. T. Elliott, was inclined to consider it of parasitic origin.

In another case ergot not only failed entirely, but the patient became affected with a furuncular condition, which aggravated sufferings produced by the chronic eczema. This use of ergot is not mentioned in any of the works above mentioned.

The New York Dermatological Society recently voted almost unanimously against the use of vaseline in skin diseases, it being said to be often irritating, to interfere with absorption of drugs and to possess other undesirable properties. Dr. G. H. Fox relates his gratifying experience in the use of chloroform or ether in the form of spray upon an urticarial eruption, paleness and relief of itching and burning following. He says he has forgotten whether chloroform or ether spray acted best. Some of the German skin specialists mention evaporating "sprays" as beneficial in hyperæmic or inflamed conditions of the skin, alcohol being especially mentioned, I believe, though Hebra also speaks of the addition of ether or chloroform. Crocker refers to the employment of citric acid in chloroform water as useful in urticaria.

Plaster containing 20 per cent. acid salicylic has been seen to act well as a "keratolytic" on conditions of "hyperkeratosis."

Dr. G. T. Elliott, one of the "clinic physicians," follows the German plan of treating tubercular or gummatous syphilides locally alone with mercury. The effect has been very gratifying in the majority of cases. Robinson favors, in his work, a combined local and internal treatment. A woman at the Hospital had a tubercular syphilide near the right eye which was circular, tending to resolution on one side and to advancement on the opposite; infiltration present but no ulceration. Another characteristic, tertiary lesion near angle of mouth. No internal treatment, but Ungt. Hg. was ordered applied. In a week the affected points were scarcely visible.

Aq. piper. menth. has been recommended as relieving *prunus*

*vulvæ*, and the result in a case at the S. and C. Hospital, seems to justify its employment. Part affected with a general acute eczema, involving the pudenda also, where itching was intense. Instead of the water previously used in her lotion (calamine, zinc, etc.,) peppermint water was used. Itching was relieved, but of course the disease remained, and was, perhaps, even a little aggravated by the lotion.

At the Academy of Medicine, the evening of June 6th, Dr. P. A. Morrow read a paper upon leprosy, and described the territory it occupied, the characteristics of the disease, and dwelt especially upon the contagiousness but non-heredity of the disease. The object of the paper was to interest, primarily, the profession in taking steps towards legislation for the isolation of persons affected with the disease and prevention of its spread. Late in the winter Dr. Morrow made a trip to the Sandwich Islands, taking in Louisiana and Mexico *en route*, enquiring at points along the journey, and studying the disease where present in California. He was unable to study the disease in the leper settlement in Louisiana, as the victims have become sensitive and retiring because of frequent newspaper articles about them. In Mexico lepers are quite numerous, the anæsthetic variety of the disease predominating. No especial efforts are made to isolate the cases. At points in Texas he learned of existing cases. In California he obtained photographs of several cases, two or three being in the negro race. At Molokai, in the Sandwich Islands, is situated the leper settlement, where all lepers who are discovered by the Board of Health must go. Relatives of the patients are permitted to accompany them as nurses. Dr. M. obtained a number of photographs of the various types and stages of the disease, and at the meeting above mentioned showed some of them by means of a "magic lantern." The tubercular type is greatly predominant in the Sandwich Islands. Dr. Morrow holds that the tubercular type is alone contagious. Does not believe in hereditary leprosy, but is convinced of its contagiousness. He, however, showed the photograph of a man who had seen three wives in succession go to the leprosy settlement, while he seemed to remain entirely free of the disease.

One peculiar symptom which he mentioned was an ulceration in the sole of the foot characteristic of the disease, and so well known that lepers who wish to escape being sent away conceal this if possible. The doctor mentioned India, China, Japan, Europe, Brazil, the West Indies, Mexico, and the United States and Canada, each as having areas of leprosy infected territory. He illustrated these by maps upon which these areas were marked in red shading, proportional to the percentage. Only one case in the world is known, or positively believed to have been successfully inoculated with the bacilli of the disease (which, it is said, have been definitely isolated) and this man was seen and photographed by Dr. Morrow.

In 1884, this man was under sentence of death, and being given choice between this penalty and that of being inoculated with leprosy and kept in prison for life, he chose the latter. For a long time after the inoculation he remained entirely free, but has now developed characteristic "tubercles" of the disease, which now will be seen in the photograph.

Dr. M. stated that the belief in the contagiousness of the disease is gaining supporters, though at one time medical men were inclined to doubt such a property. In the discussion, prominent dermatologists and venereal specialists, as Drs. Allen, Bulkley, Fox, Sturgis, Pifford and Taylor, expressed their views. Dr. Allen was inclined to the "contagiousness" view; Dr. Bulkley believed something should be done to prevent a spread of the disease in this country; Dr. Fox did not seem to think there was any danger, and Dr. Sturgis disputed the view that there were not the conditions present which favor its development here. Dr. Pifford mentioned having brought the same subject to the attention of the Academy ten years ago and having suggested steps for checking growth of the disease. Dr. Taylor seemed to consider "the danger" trivial.

It may be of interest to state that there has been a case in Charity Hospital for four or five years. There is no evidence that any other case has developed there. A case is also said to be in the German Hospital, and there is one at the Country Branch of the Skin and Cancer Hospital, but he is isolated, which was not done in the case of the man at Charity Hospital.

M. B. HUTCHINS, M. D.

